# Minimally Invasive Sutureless Aortic Valve Replacement With the Perceval S Bioprosthesis Through Ministernotomy: A Single-Center Experience

**DOI:** 10.7759/cureus.11212

**Published:** 2020-10-28

**Authors:** Krishan Ramsaransing, Vikash Hindori, Athiná Kougioumtzoglou, Abdullah Kaya, Eva Verbeek

**Affiliations:** 1 Cardiothoracic Surgery, Onze Lieve Vrouwe Gasthuis Ziekenhuis (OLVG) Amsterdam, Amsterdam, NLD; 2 Cardiothoracic Surgery, Amsterdam University Medical Center, Amsterdam, NLD

**Keywords:** aortic valve surgery, minimally invasive cardiac surgery, suture free

## Abstract

Objectives

Minimally invasive aortic valve replacement has the potential advantage of faster postoperative recovery compared to open procedures. Moreover, aortic valve replacement with a sutureless valve shortens procedure time. The aim of this study is to report early postoperative outcomes and one-year survival of patients undergoing sutureless aortic valve replacement with the Perceval S bioprosthesis (LivaNova, Milan, Italy) through a ministernotomy.

Methods

A total of 110 patients underwent sutureless aortic valve replacement in our center with the Perceval S bioprosthesis through a ministernotomy between February 2016 and March 2019. Data regarding preoperative and operative details, hospital stay, postoperative outcomes within 30 days after surgery, and one-year mortality were assessed.

Results

The mean cross-clamping time and extracorporeal circulation time were 54 ± 14 and 78 ± 21 minutes, respectively. No conversion to full median sternotomy was needed perioperatively. In-hospital mortality was 0.9%. Postoperative peak gradient was 13.3 mmHg; no major paravalvular leakage or valve migration occurred postoperatively. Postoperative complications consisted of one (0.9%) patient requiring full sternotomy for bleeding and two (1.8%) patients requiring re-ministernotomy due to acute tamponade. Pacemaker implantation was needed in four (3.6%) patients. Postoperative ischemic stroke rate and new-onset atrial fibrillation were 0.9% (n = 1) and 20% (n = 22), respectively, and one-year survival was 97.3%. Median intensive care unit and hospital stay were one and eight day(s), respectively.

Conclusion

Minimally invasive sutureless aortic valve replacement with the Perceval S bioprosthesis through a ministernotomy appears to be a safe procedure with good postoperative results and one-year survival. Further follow-up is needed to evaluate long-term outcomes.

## Introduction

Severe aortic valve stenosis is one of the most common valve diseases in the Western world and is characterized by calcification and thickening of the valvular leaflets. It is a progressive disease that can lead to heart failure and death from cardiovascular causes. The prevalence of aortic valve stenosis is increasing due to an aging population: in patients between the ages of 60 and 69, the prevalence is 1.3%, but this increases to 9.8% in octogenarians [1].

Currently, there are no medications that have been proven to attenuate or reverse calcification of the valve, leaving aortic valve replacement (AVR) as the only available treatment [2,3].

Surgical AVR (SAVR) remains the golden standard, with perioperative mortality rates that have progressively decreased over the years to approximately 1-3% [3,4].

Although excellent results have been shown for SAVR, it is an invasive procedure that requires median sternotomy for access to the heart. For high-risk patients, for instance, patients with low ejection fraction, high age, pulmonary or neurological dysfunction, this conventional method may lead to poor clinical outcomes [5,6].

However, over the last decade, minimally invasive AVR (MIAVR) techniques have evolved as an alternative for full median sternotomy.

In MIAVR, a smaller incision, either partial sternotomy or a J-shaped upper ministernotomy, is used, which has the potential advantage of faster postoperative recovery, less pain, and shorter hospital stay [7,8]. A potential disadvantage of MIAVR is that it is associated with longer procedural duration, which poses a potential risk to patients [9]. The use of sutureless aortic valve prostheses allows for shorter cross-clamp and cardiopulmonary bypass (CPB) times due to faster implantation. Several studies report promising results with the sutureless Perceval S bioprosthesis (LivaNova, Milan, Italy) [10-12].

The aim of this study was to report early postoperative outcomes and one-year survival of patients undergoing sutureless AVR with the Perceval S bioprosthesis through an upper ministernotomy.

## Materials and methods

Patients and methods

The Institutional Ethics Committee was asked for approval of this retrospective study, and the need for informed consent was waived.

From February 2016 to March 2019, a total of 110 patients (52 males; mean age: 74.2 ± 6.1 years) with severe symptomatic aortic valve stenosis underwent sutureless AVR in our center with the Perceval S bioprosthesis through a ministernotomy.

Inclusion criterion was patients of 18 years or older with AVR by means of ministernotomy using a Perceval S bioprosthesis. In our study, the European Society of Cardiology (ESC) Guidelines for the Management of Valvular Heart Disease were followed. Exclusion criterion was patients with comorbidity requiring a concomitant procedure, emergency intervention, porcelain aorta, active endocarditis and an irregular aortic annulus or ascending aorta anatomy, the use of another type of aortic valve prosthesis, or full median sternotomy.

All patients had preoperative screening with a transthoracic echocardiographic study for a better evaluation of the aortic valve and estimation of the gradients, aortic annulus diameter, and symmetry of the Valsalva sinuses.

A computed tomography (CT) scan was performed to determine the distance and location between the aorta and the chest wall.

The operative risk of these patients was estimated according to the European System for Cardiac Operative Risk Evaluation (EuroSCORE) II. Median EuroSCORE II was 1.45% (interquartile range: 0.93%).

The 30-day mortality was calculated, and overall one-year survival was established during follow-up.

Postoperative transthoracic echocardiography was performed before discharge to measure gradients and paravalvular leakage (PVL). The grade of PVL was classified into four grades: trivial, mild, moderate, and severe.

Complications until discharge were assessed, including re-exploration for bleeding, perioperative stroke, perioperative myocardial infarction, total atrioventricular (AV) block, pacemaker implantation due to complete AV block, postoperative atrial fibrillation, and infective complications. Intensive care unit (ICU) stay and total length of stay were assessed.

Surgical technique

The Perceval S bioprosthesis is a biologic aortic valve prosthesis and consists of a tissue component made from bovine pericardium and a self-expandable nitinol stent, which has the dual role of supporting the valve and fixing it in place.

The ministernotomy was achieved through a 4-cm midline vertical skin incision performing a partial J sternotomy at the third intercostal space. All surgical procedures were performed by either one of two experienced surgeons in MIAVR with the sutureless technique. Standard CPB was established by arterial cannulation of the ascending aorta and venous cannulation of the right femoral vein. Venting was achieved through placement of a 12-Fr catheter in the pulmonary trunk, and cardioplegic arrest was induced after aortic cross-clamp. A transverse aortotomy was performed at the level of the epiaortic fat pad. After decalcification and removing of the diseased valve, sizing took place with a valve sizer to estimate the appropriate valve size. Three guiding sutures were placed 1 or 2 mm below the nadir cusp for accurate alignment of the inflow portion of the new valve into the aortic annulus. Subsequently, the Perceval bioprosthesis was implanted, ballooning was performed, guiding sutures were removed, the aortotomy was closed, and the patient was weaned from CPB. The valve was evaluated for function and PVL with transesophageal echocardiography before decannulation.

Statistical analysis

Continuous data were checked for normality by performing a Shapiro-Wilk and/or Kolmogorov-Smirnov test. Normally distributed data are presented as mean and standard deviation. Non-normally distributed data are presented as median and interquartile range (IQR), and categorical data are presented as percentages. Linear regression was used to evaluate change in duration of mean cross-clamping and CPB times over the study period. All statistical analysis was performed using SPSS Statistics for Windows, Version 22.0 (IBM Corp., Armonk, NY, USA).

## Results

In total, 110 patients were included in our study. Baseline patient characteristics are reported in Table 1.

**Table 1:** Baseline Patient Characteristics (N = 110) Data are presented as N (%), mean ± standard deviation, or median ± interquartile range. eGFR, estimated glomerular filtration rate; EuroSCORE, European System for Cardiac Operative Risk Evaluation; NYHA, New York Heart Association

Baseline Characteristics	Value
Age (years)	74.2 ± 6.1
Male	52 (47.3%)
Weight (kg)	81.2 ± 14.4
Height (cm)	168.7 ± 9.1
Body mass index	28.5 ± 5
Hypertension	56 (50.9%)
Chronic obstructive pulmonary disease	14 (12.7%)
Diabetes mellitus	40 (36.4%)
Extracardiac arteriopathy	6 (5.5%)
Prior cerebrovascular accident	2 (1.8%)
Recent myocardial infarction	2 (1.8%)
Moderate left ventricle function (ejection fraction 30-50%)	7 (6.4%)
Chronic renal failure (eGFR < 60)	16 (14.5%)
NYHA class III/IV	18 (16.3%)
EuroSCORE II	1.45 ± 0.93
Preoperative peak gradient (mmHg)	74.6 ± 21.1
Preoperative aortic valve area (cm^2^)	0.8 ± 0.19

The mean cross-clamping time was 54.0 ± 13.7 minutes, and the mean CPB time was 77.8 ± 21.2 minutes (Table 2). Linear regression showed an 8.2% decrease over time (4.6 minutes) in mean cross-clamping time and a 13.3% decrease (11.1 minutes) over time in mean CPB time. No conversion to full median sternotomy was needed intraoperatively. The incidence of bicuspid valves (Sievers type 1) was 13.6% (15 patients). Perceval size S was implanted in 15 (13.6%) patients, Perceval size M was implanted in 26 (23.6%) patients, L in 42 (38.2%) patients, and XL in 27 (25.5%) patients.

**Table 2:** Peroperative Characteristics Data are presented as mean ± standard deviation or N (%) CPB, cardiopulmonary bypass

Perioperative Characteristics	Value
Cross-clamp time (minutes)	54.0 ± 13.7
CPB time (minutes)	77.8 ± 21.2
Conversion to sternotomy	0
Bicuspid valve	15 (13.6%)
Perceval size S	15 (13.6%)
Perceval size M	26 (23.6%)
Perceval size L	42 (38.2%)
Perceval size XL	27 (24.5%)

No major PVL or valve migration occurred postoperatively. Only trivial PVL was assessed in two (1.8%) patients. Postoperative peak and mean gradients were 21.6 ± 9.62 mmHg and 13.3 ± 4.7 mmHg, respectively.

Mean ICU stay in hours was 34 ± 51 minutes, median ICU stay in days was one day (range: 1-21 days), and median hospital stay was eight days (range: 5-80 days).

Re-exploration with a full median sternotomy was needed in one patient due to bleeding. Postoperative acute tamponade was seen in two (1.8%) patients, and in these cases, full median sternotomy was needed. No sternal infections were seen postoperatively. A definitive pacemaker implantation as a result of complete heart block was needed in four (3.6%) patients. Postoperative ischemic stroke rate, assessed by a neurologist, was 0.9% (N = 1), and postoperative new-onset atrial fibrillation was 20% (N = 22) (Table 3). One (0.9%) patient had an uncomplicated urinary tract infection, and three (2.7%) patients had uncomplicated pneumonia.

**Table 3:** Postoperative Complications AV, atrioventricular

Complications	N (%)
Postoperative stroke	1 (0.9%)
Bleeding	1 (0.9%)
Resternotomy	3 (2.7%)
Pacemaker implantation due to total AV block	4 (3.6%)
New-onset postoperative atrial fibrillation	22 (20%)

Hospital mortality was 0.9% (N = 1). This patient died of pneumonia due to Haemophilus influenzae infection complicated by irreversible diastolic cardiac failure. The 30-day mortality was 1.8% (N = 2). The cause of death in one patient was sepsis due to endocarditis, and the cause of death in the other patient was unknown.

One-year survival was 97.3%. Mean follow-up in our study was 2.69 years. For those patients for whom three-year follow-up was available, the three-year survival was 96.3%.

## Discussion

The aim of this study is to report our early postoperative outcomes and one-year survival of patients undergoing sutureless AVR with the Perceval S bioprosthesis through a ministernotomy. In MIAVR, a smaller incision is required than in conventional AVR, which potentially results in a less painful and faster postoperative recovery period and therefore shorter hospitalization. Using a sutureless valve allows the surgical procedure to be shortened due to faster implantation [7].

The results in our study are largely consistent with previous studies which show that a minimally invasive approach combined with sutureless implantation technique results in shorter procedural time, better clinical outcomes, and low mortality [11,13-15]. For instance, 30-day mortality was 1.8% in our study, whereas it was 2.1% [11] and 2.4% [15] in previous studies. Although we have not assessed hospital costs in our study, previous studies show reduced hospital costs as a result of lower rate of postoperative complications, reduced resource consumption, and reduced hospital stay [13]. However, the CPB and cross-clamping times are longer in our study when compared to previous studies [14,15]. These longer procedure times could be a side effect of the recently introduced techniques, requiring a learning curve for the surgeon with regard to both the ministernotomy and sutureless bioprosthesis implantation. This is also reflected in the results of linear regression of cross-clamping and CPB duration. A significant decrease is witnessed over time, suggesting that more experience using these techniques leads to shorter cross-clamping and CPB duration.

No sternal wound infections occurred in this cohort. This could be due to our small sample size (N = 110), but the small size of the ministernotomy may also have contributed to this outcome.

Complete heart block requiring a permanent pacemaker is a known complication of AVR, especially in association with sutureless AVR, due to radial forces required to keep the prosthesis in place [16]. A 2018 meta-analysis on 639 Perceval S implants showed pacemaker implantation rates of 7.9%, which was significantly higher than pacemaker implantation rates where a conventional bioprosthesis was used [17]. The incidence of complete heart block requiring permanent pacemaker in our study was 3.6%, which is low compared to other studies in which the use of the Perceval valve was researched [14,18]. However, it is not unique, as results from an international prospective study recently demonstrated pacemaker implantation in 3.3% [19]. Adequate surgical precautions (adequate sizing of valve and placement of guiding sutures) could have contributed to the lower incidence of postoperative permanent pacemaker implantation. In addition, in our postoperative protocol, a five-day observation period was used to detect conduction disorders. Two patients had temporary total AV block, most likely caused by postoperative edema. These patients did not require permanent pacemaker implantation, as their total AV block subsided within our observation period.

Median ICU stay was one day (range: 1-21 days), and median hospital stay was eight days (range: 5-80 days). Although studies demonstrated a shorter hospitalization in MIAVR when compared to conventional AVR, this is not reflected in our study [20]. However, the rhythm observation period of five days embedded in our postoperative protocol contributed to the longer duration of ward stay in our study. We found no major PVL in our study. This could be attributed to our careful patient selection, echocardiographic assessments, and correct intraoperative sizing.

The limitations of the present study are its retrospective nature and the lack of a control group and randomization. In addition, the study is limited by a small sample size and based on cases from a single center. Even though both the ministernotomy and sutureless valves techniques were newly introduced and therefore subject to a learning curve from the surgeon, the majority of surgeries were performed by a single surgeon. Therefore, although the sample size is limited, variance in outcomes due to surgical proficiency may be reduced compared to multi-center studies. Ideally, for future research, sample size should be increased and follow-up should contain long-term evaluation of valve function.

## Conclusions

Minimally invasive sutureless AVR with the Perceval S bioprosthesis through a ministernotomy appears to be a safe procedure with good postoperative results and one-year survival. Further follow-up is needed to evaluate more long-term outcomes.
